# The Impact of Diabetes on the Prognosis of Upper Tract Urothelial Carcinoma After Radical Nephroureterectomy: A Systematic Review and Meta-Analysis

**DOI:** 10.3389/fonc.2021.741145

**Published:** 2021-10-18

**Authors:** Xiaoshuai Gao, Liang Zhou, Jianzhong Ai, Wei Wang, Xingpeng Di, Liao Peng, Banghua Liao, Xi Jin, Hong Li, Kunjie Wang

**Affiliations:** Department of Urology, Institute of Urology (Laboratory of Reconstructive Urology), West China Hospital, Sichuan University, Chengdu, China

**Keywords:** upper tract urothelial carcinoma, diabetes, radical nephroureterectomy, prognosis, meta-analysis

## Abstract

**Background:**

Studies have reported that diabetes is related to the prognosis of upper tract urothelial carcinoma (UTUC) after radical nephroureterectomy (RNU), but this conclusion is still controversial. Here, we performed a meta-analysis to comprehensively explore the association between diabetes and UTUC prognosis.

**Methods:**

In November 2020, we searched PubMed, Web of science and the Cochrane Library to find relevant studies that evaluated the effect of diabetes on the prognosis of UTUC. The Newcastle Ottawa Scale was used to assess the quality of the literature. Review Manager 5.3 was used to pool cancer-specific survival (CSS), overall survival (OS), recurrence-free survival (RFS) and intravesical recurrence (IVR).

**Results:**

A total of 10 studies with 11,303 patients were included in this meta-analysis. Our pooled results showed that diabetes did not affect the survival outcome of UTUC, including CSS (HR: 1.33, 95% CI: 0.89-1.98; *P* = 0.16), OS (HR: 1.18, 95% CI: 0.77-1.80; *P* = 0.45) and RFS (HR: 1.37, 95% CI: 0.91-2.05; *P* = 0.13). However, diabetes increased the risk of IVR of UTUC patients (HR: 1.26, 95% CI: 1.11-1.43; *P* = 0.0004).

**Conclusion:**

Although diabetes has no significant impact on the survival outcomes of UTUC after RNU, it increases the risk of IVR. Therefore, special attention should be paid to monitoring the IVR for UTUC patients with diabetes and the necessity of appropriate intravesical adjuvant treatment when needed.

## Introduction

Upper tract urothelial carcinoma (UTUC) is a rare cancer with a yearly incidence of only 1 to 2 cases per 100,000 individuals (1). Although the incidence of UTUC is low, it is always malignant and locally invasive (2). Therefore, radical nephroureterectomy (RNU) is the current gold standard for UTUC treatment (3). Even after RNU, the risk of UTUC recurrence and related mortality is still high. The five-year overall mortality rate in UTUC patients treated with RNU is approximately 40%, and the cancer-specific mortality rate is as high as 25% (3, 4). Therefore, it is necessary to study the prognostic factors of UTUC.

Current prognostic models are based on preoperative factors like tumor multifocality, size, location, tumor stage and grade on biopsy, hydronephrosis (5, 6) and postoperative predictors such as T stage, lymphovascular invasion, tumor necrosis, architecture and concomitant carcinoma in situ (5, 7). These prognostic factors classified UTUC as low risk or high risk and determined whether the patients should undergo kidney-sparing procedures or RNU. Additionally, these models are very important for predicting the survival outcomes of UTUC patients and whether they will be treated with adjuvant chemotherapy after surgery (8). However, these predictive models have limited their clinical applications due to low accuracy. Therefore, it is necessary to find new predictive factors for UTUC patients to increase the accuracy of prognostic models.

In recent years, the incidence of diabetes has increased with changes in diet and lifestyle. Moreover, there are studies showing that diabetes increases the risk of cancer of the liver, colorectum, breast, endometrium and pancreas (9). In addition, diabetes is also an important risk factor for bladder cancer and prostate cancer (10, 11). To date, several studies have also shown that diabetes is related to UTUC prognosis, but this conclusion is still controversial. We aimed to explore the effects of diabetes on the prognosis of UTUC through this meta-analysis and draw conclusions with a stronger evidence to guide clinical practice of UTUC treatment.

## Materials and Methods

### Search Strategy

Two researchers searched PubMed, Web of science and the Cochrane Library in November 2020 according the PRISMA guidelines (12). They searched for studies on diabetes and the prognosis of UTUC using the following key terms: (“diabetes” OR “glycemic”) AND (“upper tract urothelial carcinoma” OR “upper tract urinary carcinoma”) AND (“survival” OR “prognostic” OR “progression” OR “recurrence”).

### Inclusion and Exclusion Criteria

We developed the inclusion and exclusion criteria according to the populations, interventions, comparators, outcomes, and study designs (PICOS). Studies were considered eligible if they met the following selection criteria: UTUC patients (populations) after RNU (interventions), studies evaluating the association of diabetes (comparators) with UTUC prognosis (outcomes). All prospective or retrospective controlled trials (study designs) were included in this meta-analysis. The exclusion criteria included the following items: reviews, case series reports, abstract and letters. The study did not include any indicator and its corresponding HR and 95% intervals of cancer-specific survival (CSS), recurrence-free survival (RFS), overall survival (OS) or intravesical recurrence (IVR). In cases of repeated publications, only the largest publication was included.

### Data Extraction and Quality Assessment

Two researchers (GXS and WW) independently extracted data from the included articles. Any disagreements were resolved by consulting a third researcher (DXP). The following variables were extracted: first author, publication time, study period, patient number, patient age and sex ratio, follow-up time, number of diabetes cases, recurrence, mortality, cancer-specific mortality, adjuvant chemotherapy, and HR and 95% intervals of CSS, RFS, IVR and OS. The Newcastle Ottawa Scale (NOS) was used to evaluate the quality of the selected studies (13).

### Statistical Analysis

For CSS, RFS, IVR and OS, we pooled the HRs with 95% CIs to evaluate the effect of diabetes on UTUC. Q test and I^2^ test were used to evaluated the heterogeneity among studies. The random effects model was performed for high heterogeneity among the outcomes by P<0.05 or I^2^ >50%. Otherwise, the fixed effects model was used. We evaluated publication bias by funnel plots. Sensitivity analysis was evaluated by removing a single study to assess the stability of the meta-analysis. All statistical analyses were performed using Review Manager software version 5.3.

## Results

### Literature Screening and Quality Assessment

**Figure 1:**
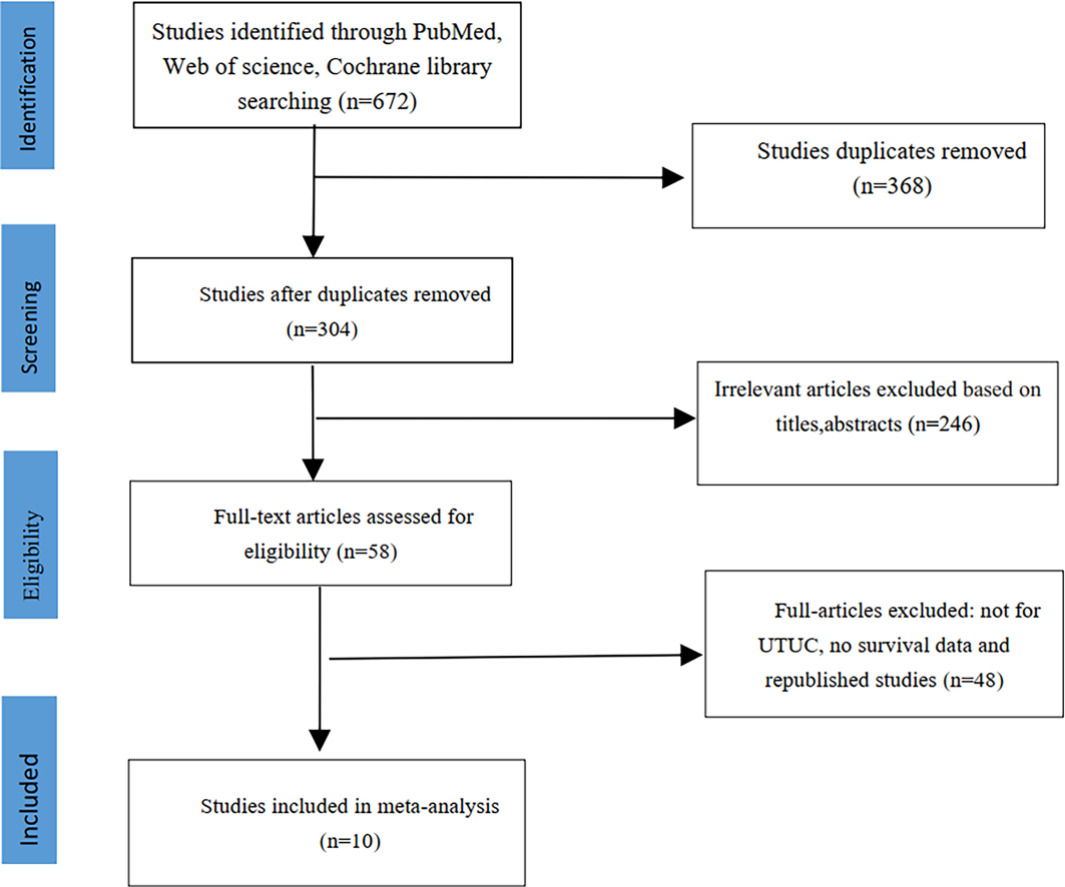
The flowchart showing study search and selection process.

We initially identified 672 studies from the three databases mentioned above. Ten studies were ultimately included in this meta-analysis based on our inclusion and exclusion criteria (14–23). A total of 12,865 patients had UTUC, and 2045 (15.9%) had diabetes. The literature screening process is shown in **Figure 1**. The clinical characteristics of the included studies are listed in **Table 1**. The median age of patients in all included studies was over 60 years old. All included studies were retrospective controlled trials, with NOS scores ranging from 7 to 8. The details scores are listed in **Table 1**.

**Table 1:** Basic characteristics and data of included articles.

Author and year	Region	Period	Sample size	Median age, years	Female n (%)	Tumor location			Follow-up (median month)	Recurrence	Mortality	CSM	AC	Study type	NOS score	Diabetes
						Renal pelvic	Ureter	Multifocal								
Jeon and Tae (18)	Korea	2003-2016	176	Mean=66.1	53 (30.1%)	66	82	26	Mean=36.9	N	N	N	42	CCT	7	42
Bao et al. (14)	China	1999-2015	754	69.0	412 (54.6%)	N	N	N	61	N	234	165	N	CCT	7	146
Xu et al. (23)	China	2003-2016	644	67.5	277 (43.0%)	338	191	115	39	269	233	185	270	CCT	8	223
Fang et al. (16)	China	1999-2011	612	68.0	272 (44.4%)	341	271	N	64	206	210	187	N	CCT	8	101
Lin et al. (20)	Taiwan	2002-2007	5141	Mean=67.3	2779 (54.1%)	N	N	N	22.4	1837	1796	N	N	CCT	7	863
Huang et al. (17)	China	2003-2013	425	67.0	146 (34.4%)	208	137	80	38.5	166	147	106	84	CCT	7	53
Qin et al. (21)	China	2012-2016	346	Mean=66.6	140 (40.4%)	175	171	N	21	52	40	N	169	CCT	7	68
Kang et al. (19)	Korea	2004-2014	566	70.0	165 (29.2%)	258	308	N	33.8	53	44	N	205	CCT	8	135
Rieken et al. (22)	multination	1987-2007	2492	69.2	811 (32.5%)	1613	879	590	36	663	884	545	247	CCT	7	365
Cho et al. (15)	Korea	2004-2012	147	70.0	106 (72.1%)	74	73	N	33	N	N	N	95	CCT	7	49

NOS, Newcastle-Ottawa Scale; CCT, retrospective case control study; CSM, Cancer-specific mortality; AC, Adjuvant chemotherapy; N, not available.

### Survival Outcomes

**Figure 2:**
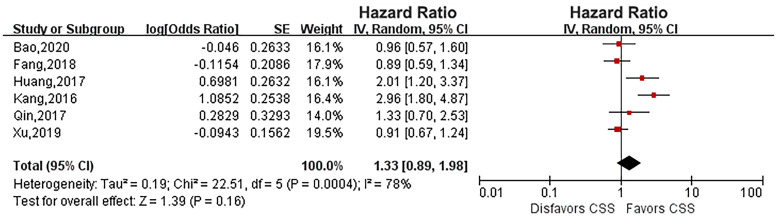
Forest plot of the impact of diabetes on cancer-specific survival.

**Figure 3:**
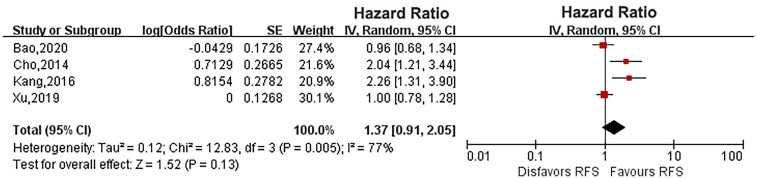
Forest plot of the impact of diabetes on recurrence-free survival.

**Figure 4:**
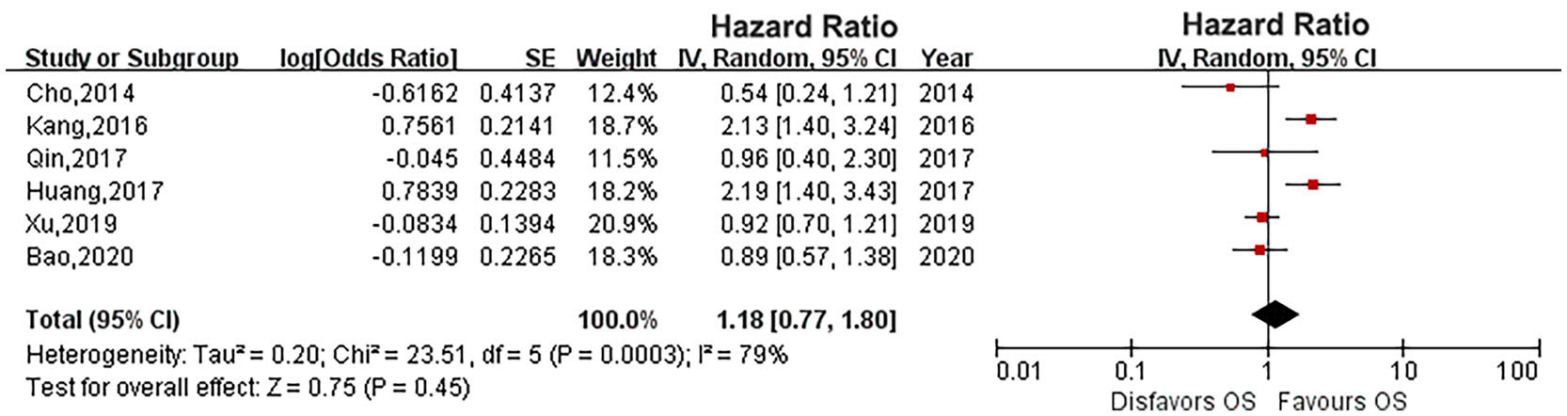
Forest plot of the impact of diabetes on overall survival.

Six eligible studies evaluated the impact of diabetes on CSS in UTUC (14, 16, 17, 19, 21, 23). As the heterogeneity among studies was high, a random effects model was used (*P* = 0.0004, *I* ^2^ = 78%). Pooled results showed that there was no relationship between diabetes and CSS (HR: 1.33, 95% CI: 0.89-1.98; *P* = 0.16; **Figure 2**). Four eligible studies (14, 15, 19, 23) evaluated the impact of diabetes on RFS, and the meta-analysis revealed that diabetes did not affect RFS (HR: 1.37, 95% CI: 0.91-2.05; *P* = 0.13; **Figure 3**). Six studies provided data regarding the OS between the two groups (14, 15, 17, 19, 21, 23). As high heterogeneity existed among the studies (*P* = 0.0003, *I* ^2^ = 79%), we used the random effect model, which showed that the OS was similar between the two groups (HR: 1.18, 95% CI: 0.77-1.80; *P* = 0.45; **Figure 4**).

### Intravesical Recurrence

**Figure 5:**
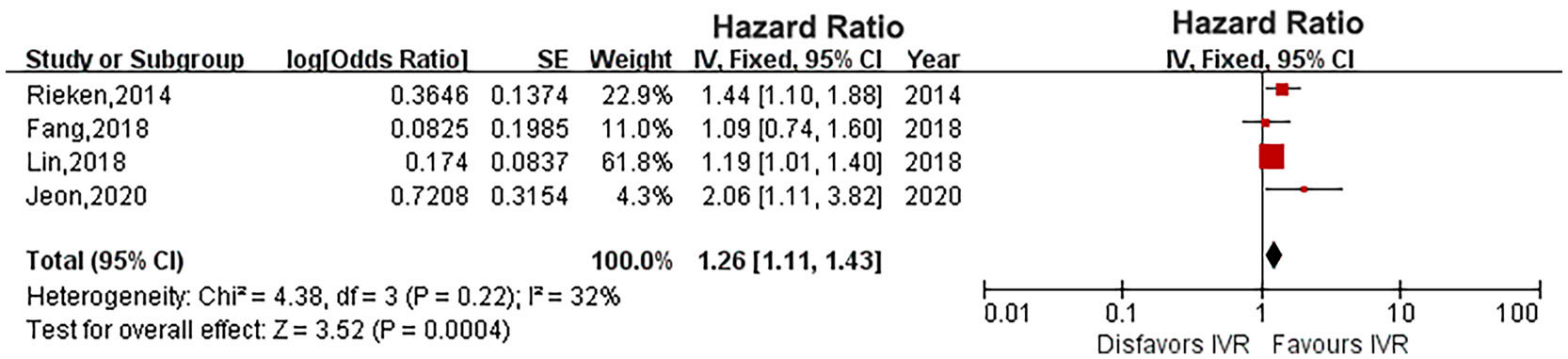
Forest plot of the impact of diabetes on intravesical recurrence.

Four studies provided IVR data (16, 18, 20, 22). The heterogeneity was low (*P* = 0.22, *I* ^2^ = 32%), and a fixed-effects model showed that diabetes significantly increased the risk of IVR in UTUC (HR: 1.26, 95% CI: 1.11-1.43; *P* = 0.0004; **Figure 5**).

### Sensitivity Analysis and Publication Bias

A funnel plot was used to evaluate publication bias, and no evidence for publication bias was found (**Supplementary Figure 1**). Sensitivity analysis was performed by removing a single study to assess the stability of the meta-analysis. After removing a single study, the pooled HR and heterogeneity did not change significantly, and no source of heterogeneity was found.

## Discussion

Approximately one-third of UTUC patients will experience early recurrence after RNU, and nearly 80% of these patients will eventually die due to cancer (24). The current prognostic models are helpful to the selection of surgical methods and the prediction of survival outcomes of UTUC patients, but the accuracy is insufficient (8). Several studies have reported that diabetes may be one of the potential predictors of postoperative survival outcomes in UTUC patients (14, 16, 17, 19, 21, 23). Therefore, we firstly performed a meta-analysis to evaluate the impact of diabetes on the prognosis outcomes in UTUC patients after RNU. We found that diabetes did not affect any postoperative survival outcomes in UTUC patients. However, it increased the risk of IVR in UTUC. This result implies the importance of paying extra attention to IVR of UTUC patients after RNU and the necessity of appropriate intravesical adjuvant treatment when needed. Moreover, it is recommended that diabetic patients should well-controlled glycemia of UTUC after RNU.

The biological mechanism of IVR in RNU patients is not well understood. At present, there are two widely accepted theories of bladder tumor recurrence: panurothelial field defects and a single transformed cell after descendant intraluminal seeding (25). European Association of Urology guidelines suggest that early intravesical instillation of pirarubicin or mitomycin C after RNU for UTUC could reduce the risk of IVR (26). Therefore, it is particularly important to identify the risk factors for IVR and to intervene in UTUC patients. High-quality research shows that tumor-specific indicators such as multifocality, necrosis, ureteral location, positive preoperative urinary cytology, invasive pT stage and patient-specific predictors such as previous bladder cancer, male sex, and preoperative chronic kidney disease increase the risk of IVR in UTUC patients (25). A recent multicenter study showed that risk factors such as transurethral resection of the bladder cuff, ureteroscopic biopsy, and positive surgical margins increase IVR for UTUC after RNU (27). We found that diabetes increased the risk of IVR, which is a good supplement to the risk factors for IVR in UTUC patients.

Four studies in our systematic review compared the effect of diabetes on IVR in UTUC patients after RNU (16, 18, 20, 22). Jeon et al. (18) and Lin et al. (20) found that diabetes increases IVR in UTUC patients. Our meta-analysis supports the conclusions of Jeon et al. (18) and Lin et al. (20) showing that diabetes significantly increases IVR in UTUC patients. However, Fang et al. (16) and Rieken et al. (22) showed that the effect of diabetes on IVR is unclear. Diabetes medication may be one of the reasons for this difference. Diabetes that is not treated with metformin results in an increase in disease recurrence in UTUC patients after RNU (HR, 1.43; P=0.01), and this effect disappears after taking metformin (HR, 0.90; P=0.49) (22). In addition, the difference in tumor indicators between studies is an important factor that cannot be ignored regarding IVR in UTUC patients.

The mechanisms underlying the association of diabetes with IVR in UTUC patients are unclear. Hyperglycemia not only provides more nutrients for tumor cells but also activates insulin or insulin-like growth factor 1 (28). Insulin-like growth factor 1 promotes tumor cell proliferation and inhibits apoptosis (29). In addition, hyperglycemia affects many biological functions of cancer cells, such as proliferation, invasion, migration, and recurrence (30). The effect of hyperglycemia on urothelial cell tumors has also been extensively studied. Overexpression of insulin-like growth factor 1 in bladder urothelial cancer cells can promote proliferation and inhibit apoptosis (31). In addition, the immunity of diabetic patients is relatively low, and urinary tract inflammation will also affect the prognosis of UTUC.

Our meta-analysis included 10 retrospective studies comparing the effect of diabetes on the survival outcomes of UTUC. The results are different in these studies. Kang et al. (19) and Huang et al. (17) found that diabetes is associated with worse CSS, RFS and OS. However, Cho et al. (15), Xu et al. (23), Bao et al. (14), Qin et al. (21) and Fang et al. (16) demonstrated that there was no statistically significant difference in the effect of diabetes on UTUC survival outcomes. Our pooled results also showed that diabetes was not associated with worse survival outcomes. The different results among studies can be explained by the following factors.

Recent evidence reveals that many patients and tumor-related factors affect the prognosis of UTUC after RNU. Suzuki et al. (32) and Xu et al. (33) revealed that higher controlling nutritional status (CONUT) score, greater age, lower BMI, higher CRP, higher pT stage, higher tumor grade, higher tumor size, concomitant variant histology (CVH), no curative treatment and no usage of pembrolizumab after the diagnosis of UTUC are independent and significant adverse prognostic factors. Besides, Xu et al. (33) demonstrated that CONUT score was an independent predictor for CSS, RFS and OS. Moreover, Marcq et al. (34) performed an international collaborative study to evaluate the predictive value of a new classification of UTUC for kidney-sparing surgery. They found that the higher age, biopsy, high-grade cytology, sessile tumor, hydronephrosis and non–organ-confined disease on preoperative imaging were independently associated with muscle invasion at RNU (34).

Glycemic control for diabetes patients may affect the survival indicators of UTUC. Kang et al. (19) found that poorly controlled diabetes (no DM vs HbA1c > 7) was associated with worse CSS (HR, 2.96; P=0.001), RFS (HR, 2.26; P=0.003) and OS (HR, 2.13; P=0.001). However, there was no significant difference in the effect of diabetes on UTUC survival indicators in patients with well-controlled glycemia (no DM vs HbA1c < 7). Rieken et al. (22) also found that diabetic patients who did not treated with metformin were at significantly higher risk of disease recurrence and cancer-specific death compared to nondiabetic patients and diabetic patients who used metformin. Moreover, previous meta-analysis indicated that metformin intake was associated with an improved RFS (HR=0.55; P=0.01), increased progression-free survival (HR=0.70; P=0.03), and prolonged CSS (HR=0.57; P=0.002) (35). In fact, smoking can also interfere with the prognosis of UTUC (36). A meta-analysis shows that smoking increases recurrence and death of UTUC after RNU. Besides, Tellini et al. (37) found that smoking increased risk for major postoperative complications, infections, and mortality of patients treated with radical cystectomy for urothelial bladder cancer.

Our meta-analysis inevitably has some limitations. First, the 10 studies we included were all retrospective studies, and there may be some selection bias. Second, there were insufficient data to pool for medication use in diabetic patients (such as drug types and blood glucose levels after taking the drug). Third, the follow-up time of most studies is not long enough, which may be the reason why many differences in survival outcomes are not reflected, so it also suggests the need for longer follow-up studies. Fourth, limitations of the data in the included studies prevented analysis of interference from some factors that may affect the prognosis of UTUC, such as patient age, sex, smoking status, and drinking habits. Nevertheless, we found that diabetes can increase IVR of UTUC patients after RNU. We hope that the emergence of large-scale prospective studies in the future can further support our conclusions.

## Conclusions

Although diabetes has no significant impact on the survival outcome of UTUC after RNU, it increases the risk of IVR. Therefore, special attention should be paid to monitoring the IVR for UTUC patients with diabetes and the necessity of appropriate intravesical adjuvant treatment when needed.

## Data Availability Statement

The original contributions presented in the study are included in the article/**Supplementary Material**. Further inquiries can be directed to the corresponding authors.

## Author Contributions

XG wrote the manuscript writing. LZ collected and analyzed the data. JA, WW, and XD analyzed the data. LP, XJ, and BL helped review and revise the manuscript. HL and KW helped design the study and revise article. All authors contributed to the article and approved the submitted version.

## Funding

This study was supported by the 1.3.5 project for disciplines of excellence, West China Hospital, Sichuan University (ZYGD18011, ZY2016104 and ZYJC18015); Project of Science and Technology Department of Sichuan Province (2021YFS0116 and 2020YFS0047); Chengdu International Science and Technology Cooperation Funding (2019-GH02-00011-HZ); Project of Guangzhou Medical University (HX-H1701002).

## Conflict of Interest

The authors declare that the research was conducted in the absence of any commercial or financial relationships that could be construed as a potential conflict of interest.

## Publisher’s Note

All claims expressed in this article are solely those of the authors and do not necessarily represent those of their affiliated organizations, or those of the publisher, the editors and the reviewers. Any product that may be evaluated in this article, or claim that may be made by its manufacturer, is not guaranteed or endorsed by the publisher.

## Supplementary Material

Supplementary Figure 1 Funnel plot evaluating the publication bias. Cancer-specific survival **(A)**, recurrence-free survival **(B)**, overall survival **(C)**, intravesical recurrence **(D)**.

## References

[1] Soria F Shariat SF Lerner SP Fritsche HM Rink M Kassouf W . Epidemiology, Diagnosis, Preoperative Evaluation and Prognostic Assessment of Upper-Tract Urothelial Carcinoma (UTUC). World J Urol (2017) 35(3):379–87. doi: 10.1007/s00345-016-1928-x 27604375

[2] Chromecki TF Cha EK Fajkovic H Margulis V Novara G Scherr DS . The Impact of Tumor Multifocality on Outcomes in Patients Treated With Radical Nephroureterectomy. Eur Urol (2012) 61(2):245–53. doi: 10.1016/j.eururo.2011.09.017 21975249

[3] Ploussard G Xylinas E Lotan Y Novara G Margulis V Rouprêt M . Conditional Survival After Radical Nephroureterectomy for Upper Tract Carcinoma. Eur Urol (2015) 67(4):803–12. doi: 10.1016/j.eururo.2014.08.003 25145551

[4] Rink M Xylinas E Trinh QD Lotan Y Margulis V Raman JD . Gender-Specific Effect of Smoking on Upper Tract Urothelial Carcinoma Outcomes. BJU Int (2013) 112(5):623–37. doi: 10.1111/bju.12014 23465088

[5] Rouprêt M Babjuk M Compérat E Zigeuner R Sylvester RJ Burger M . European Association of Urology Guidelines on Upper Urinary Tract Urothelial Carcinoma: 2017 Update. Eur Urol (2018) 73(1):111–22. doi: 10.1016/j.eururo.2017.07.036 28867446

[6] Kim HS Jeong CW Kwak C Kim HH Ku JH . Association Between Demographic Factors and Prognosis in Urothelial Carcinoma of the Upper Urinary Tract: A Systematic Review and Meta-Analysis. Oncotarget (2017) 8(5):7464–76. doi: 10.18632/oncotarget.10708 PMC5352335 27448978

[7] Gao X Ma Y Chen G Chen J Li H Li H . Concomitant Carcinoma *in Situ* as a Prognostic Factor in the Upper Tract Urothelial Carcinoma After Radical Nephroureterectomy: A Systematic Review and Meta-Analysis. Urol Oncol (2020) 38(6):574–81. doi: 10.1016/j.urolonc.2020.02.020 32273049

[8] Shao Y Li W Wang D Wu B . Prognostic Value of Preoperative Lymphocyte-Related Systemic Inflammatory Biomarkers in Upper Tract Urothelial Carcinoma Patients Treated With Radical Nephroureterectomy: A Systematic Review and Meta-Analysis. World J Surg Oncol (2020) 18(1):273. doi: 10.1186/s12957-020-02048-7 33097052 PMC7585317

[9] Joost HG . Diabetes and Cancer: Epidemiology and Potential Mechanisms. Diabetes Vasc Dis Res (2014) 11(6):390–4. doi: 10.1177/1479164114550813 25268021

[10] Zhang F Yang Y Skrip L Hu D Wang Y Wong C . Diabetes Mellitus and Risk of Prostate Cancer: An Updated Meta-Analysis Based on 12 Case-Control and 25 Cohort Studies. Acta Diabetol (2012) 49 Suppl 1:S235–46. doi: 10.1007/s00592-012-0439-5 23124624

[11] Xu Y Huo R Chen X Yu X . Diabetes Mellitus and the Risk of Bladder Cancer: A PRISMA-Compliant Meta-Analysis of Cohort Studies. Medicine (Baltimore) (2017) 96(46):e8588. doi: 10.1097/md.0000000000008588 29145273 PMC5704818

[12] Moher D Liberati A Tetzlaff J Altman DG . Preferred Reporting Items for Systematic Reviews and Meta-Analyses: The PRISMA Statement. J Clin Epidemiol (2009) 62(10):1006–12. doi: 10.1016/j.jclinepi.2009.06.005 19631508

[13] Wells G Shea B O’Connell D Peterson J Welch V Losos M . The Newcasstle-Ottawa Scale (NOS) for Assessing the Quality of Non-Randomised Studies in Meta-Analysis. Available at: http://www.ohri.ca/programs/clinical_epidemiology/oxford.asp (Accessed: September 4, 2020).

[14] Bao Z Li Y Guan B Xiong G . High Preoperative Controlling Nutritional Status Score Predicts a Poor Prognosis in Patients With Localized Upper Tract Urothelial Cancer: A Propensity Score Matching Study in a Large Chinese Center. Cancer Manag Res (2020) 12:323–35. doi: 10.2147/cmar.s225711 PMC6970241 32021446

[15] Cho YH Seo YH Chung SJ Hwang I Yu HS Kim SO . Predictors of Intravesical Recurrence After Radical Nephroureterectomy for Upper Urinary Tract Urothelial Carcinoma: An Inflammation-Based Prognostic Score. Korean J Urol (2014) 55(7):453–9. doi: 10.4111/kju.2014.55.7.453 PMC4101114 25045443

[16] Fang D He S Xiong G Singla N Cao Z Zhang L . Comparison of Clinicopathologic Characteristics, Epigenetic Biomarkers and Prognosis Between Renal Pelvic and Ureteral Tumors in Upper Tract Urothelial Carcinoma. BMC Urol (2018) 18(1):22. doi: 10.1186/s12894-018-0334-7 29587736 PMC5870733

[17] Huang J Wang Y Yuan Y Chen Y Kong W Chen H . Preoperative Serum Pre-Albumin as an Independent Prognostic Indicator in Patients With Localized Upper Tract Urothelial Carcinoma After Radical Nephroureterectomy. Oncotarget (2017) 8(22):36772–79. doi: 10.18632/oncotarget.13694 PMC5482696 27906675

[18] Jeon BJ Tae BS . Preoperative Sterile Pyuria as a Prognostic Biomarker for Intravesical Recurrence in Upper Urinary Tract Urothelial Carcinoma. Investig Clin Urol (2020) 61: (1):51–8. doi: 10.4111/icu.2020.61.1.51 PMC6946823 31942463

[19] Kang SG Hwang EC Jung SI Yu HS Chung HS Kang TW . Poor Preoperative Glycemic Control Is Associated With Dismal Prognosis After Radical Nephroureterectomy for Upper Tract Urothelial Carcinoma: A Korean Multicenter Study. Cancer Res Treat (2016) 48(4):1293–301. doi: 10.4143/crt.2016.021 PMC5080827 27034146

[20] Lin MY Li WM Huang CN Lee HL Niu SW Chen LT . Dialysis Increases the Risk of Bladder Recurrence in Patients With Upper Tract Urothelial Cancer: A Population-Based Study. Ann Surg Oncol (2018) 25(4):1086–93. doi: 10.1245/s10434-017-6295-3 29330720

[21] Qin C Liang EL Du ZY Qiu XY Tang G Chen FR . Prognostic Significance of Urothelial Carcinoma With Divergent Differentiation in Upper Urinary Tract After Radical Nephroureterectomy Without Metastatic Diseases: A Retrospective Cohort Study. Medicine (Baltimore) (2017) 96(21):e6945. doi: 10.1097/md.0000000000006945 28538387 PMC5457867

[22] Rieken M Xylinas E Kluth L Trinh QD Lee RK Fajkovic H . Diabetes Mellitus Without Metformin Intake is Associated With Worse Oncologic Outcomes After Radical Nephroureterectomy for Upper Tract Urothelial Carcinoma. Eur J Surg Oncol (2014) 40(1):113–20. doi: 10.1016/j.ejso.2013.09.016 24113620

[23] Xu H Tan P Zheng X Ai J Lin T Jin X . Metabolic Syndrome and Upper Tract Urothelial Carcinoma: A Retrospective Analysis From a Large Chinese Cohort. Urol Oncol (2019) 37(4):291.e19–91.e28. doi: 10.1016/j.urolonc.2018.12.005 30584033

[24] Cha EK Shariat SF Kormaksson M Novara G Chromecki TF Scherr DS . Predicting Clinical Outcomes After Radical Nephroureterectomy for Upper Tract Urothelial Carcinoma. Eur Urol (2012) 61(4):818–25. doi: 10.1016/j.eururo.2012.01.021 22284969

[25] Seisen T Granger B Colin P Léon P Utard G Renard-Penna R . A Systematic Review and Meta-Analysis of Clinicopathologic Factors Linked to Intravesical Recurrence After Radical Nephroureterectomy to Treat Upper Tract Urothelial Carcinoma. Eur Urol (2015) 67(6):1122–33. doi: 10.1016/j.eururo.2014.11.035 25488681

[26] Rouprêt M Babjuk M Compérat E Zigeuner R Sylvester R Burger M . European Guidelines on Upper Tract Urothelial Carcinomas: 2013 Update. Eur Urol (2013) 63(6):1059–71. doi: 10.1016/j.eururo.2013.03.032 23540953

[27] Katims AB Say R Derweesh I Uzzo R Minervini A Wu Z . Risk Factors for Intravesical Recurrence After Minimally Invasive Nephroureterectomy for Upper Tract Urothelial Cancer (ROBUUST Collaboration). J Urol (2021) 206(3):568–76. doi: 10.1097/ju.0000000000001786 33881931

[28] Suikkari AM Koivisto VA Rutanen EM Yki-Järvinen H Karonen SL Seppälä M . Insulin Regulates the Serum Levels of Low Molecular Weight Insulin-Like Growth Factor-Binding Protein. J Clin Endocrinol Metab (1988) 66(2):266–72. doi: 10.1210/jcem-66-2-266 2448329

[29] Iwamura M Ishibe M Sluss PM Cockett AT . Characterization of Insulin-Like Growth Factor I Binding Sites in Human Bladder Cancer Cell Lines. Urol Res (1993) 21(1):27–32. doi: 10.1007/bf00295188 8456535

[30] Duan W Shen X Lei J Xu Q Yu Y Li R . Hyperglycemia, a Neglected Factor During Cancer Progression. Biomed Res Int (2014) 2014:461917. doi: 10.1155/2014/461917 24864247 PMC4016871

[31] Metalli D Lovat F Tripodi F Genua M Xu SQ Spinelli M . The Insulin-Like Growth Factor Receptor I Promotes Motility and Invasion of Bladder Cancer Cells Through Akt- and Mitogen-Activated Protein Kinase-Dependent Activation of Paxillin. Am J Pathol (2010) 176(6):2997–3006. doi: 10.2353/ajpath.2010.090904 20395438 PMC2877859

[32] Suzuki H Ito M Takemura K Nakanishi Y Kataoka M Sakamoto K . Prognostic Significance of the Controlling Nutritional Status (CONUT) Score in Advanced Urothelial Carcinoma Patients. Urol Oncol (2020) 38(3):76.e11–7. doi: 10.1016/j.urolonc.2019.10.014 31864938

[33] Xu H Tan P Jin X Ai J Lin T Lei H . Validation of the Preoperative Controlling Nutritional Status Score as an Independent Predictor in a Large Chinese Cohort of Patients With Upper Tract Urothelial Carcinoma. Cancer Med (2018) 7(12):6112–23. doi: 10.1002/cam4.1902 PMC6308095 30485712

[34] Marcq G Foerster B Abufaraj M Matin SF Azizi M Gupta M . Novel Classification for Upper Tract Urothelial Carcinoma to Better Risk-Stratify Patients Eligible for Kidney-Sparing Strategies: An International Collaborative Study. Eur Urol Focus (2021). doi: 10.1016/j.euf.2021.03.018 33773965

[35] Hu J Chen JB Cui Y Zhu YW Ren WB Zhou X . Association of Metformin Intake With Bladder Cancer Risk and Oncologic Outcomes in Type 2 Diabetes Mellitus Patients: A Systematic Review and Meta-Analysis. Medicine (Baltimore) (2018) 97(30):e11596. doi: 10.1097/md.0000000000011596 30045293 PMC6078654

[36] van Osch FH Jochems SH van Schooten FJ Bryan RT Zeegers MP . Significant Role of Lifetime Cigarette Smoking in Worsening Bladder Cancer and Upper Tract Urothelial Carcinoma Prognosis: A Meta-Analysis. J Urol (2016) 195(4 Pt 1):872–9. doi: 10.1016/j.juro.2015.10.139 26523878

[37] Tellini R Mari A Muto G Cacciamani GE Ferro M Stangl-Kremser J . Impact of Smoking Habit on Perioperative Morbidity in Patients Treated With Radical Cystectomy for Urothelial Bladder Cancer: A Systematic Review and Meta-Analysis. Eur Urol Oncol (2021) 4(4):580–93. doi: 10.1016/j.euo.2020.10.006 33160975

