# Supplementary figures and images for: The Impact of Diabetes on the Prognosis of Upper Tract Urothelial Carcinoma After Radical Nephroureterectomy: A Systematic Review and Meta-Analysis
Source: Front Oncol. 2021 Oct 18;11:741145. doi: 10.3389/fonc.2021.741145 (PMC8558518; doi:10.3389/fonc.2021.741145)

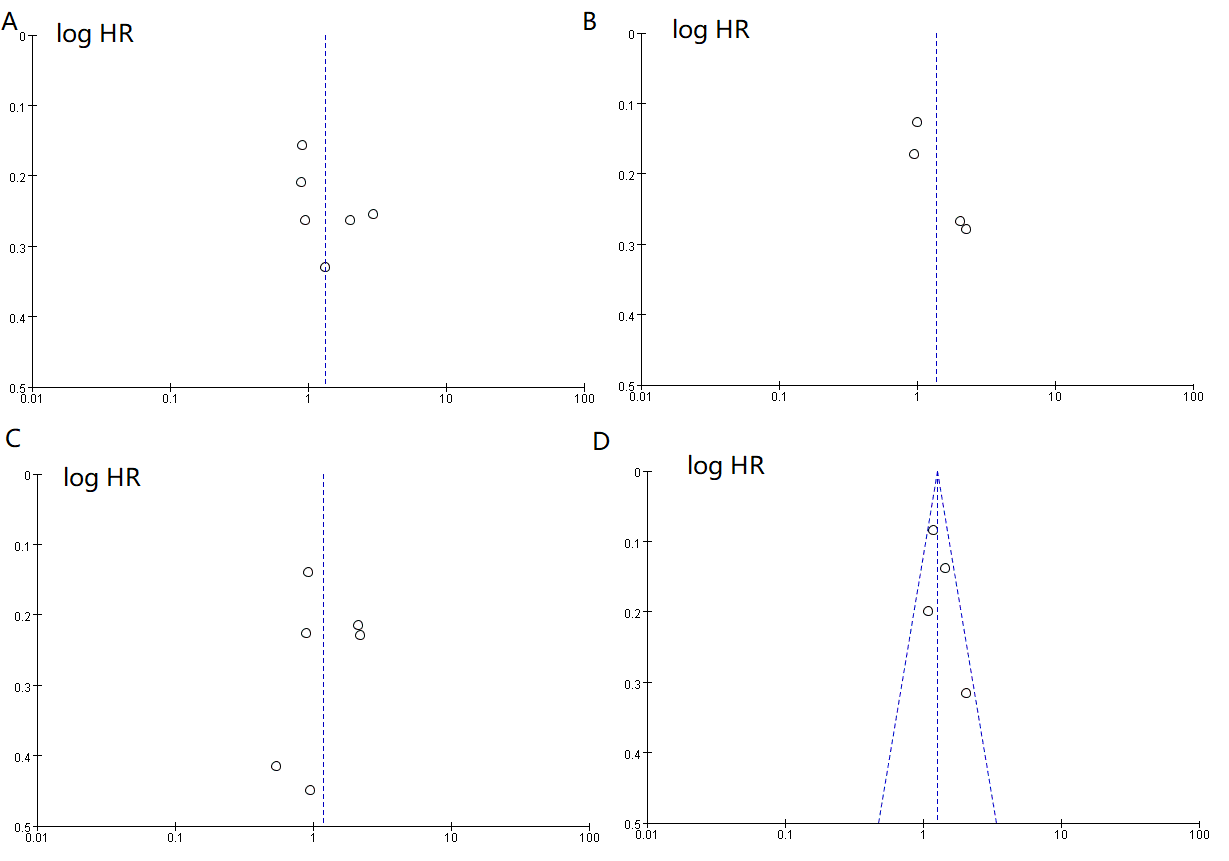

Supplement: Supplementary Figure 1 — Funnel plot evaluating the publication bias. Cancer-specific survival (A), recurrence-free survival (B), overall survival (C), intravesical recurrence (D). [file Image_1.tif]
